# Genetic Abolishment of Hepatocyte Proliferation Activates Hepatic Stem Cells

**DOI:** 10.1371/journal.pone.0031846

**Published:** 2012-02-23

**Authors:** Yoko Endo, Mingjun Zhang, Sachie Yamaji, Yong Cang

**Affiliations:** 1 Signal Transduction Program, Sanford-Burnham Medical Research Institute, La Jolla, California, United States of America; 2 Life Sciences Institute, Zhejiang University, Hangzhou, China; University of Udine, Italy

## Abstract

Quiescent hepatic stem cells (HSCs) can be activated when hepatocyte proliferation is compromised. Chemical injury rodent models have been widely used to study the localization, biomarkers, and signaling pathways in HSCs, but these models usually exhibit severe promiscuous toxicity and fail to distinguish damaged and non-damaged cells. Our goal is to establish new animal models to overcome these limitations, thereby providing new insights into HSC biology and application. We generated mutant mice with constitutive or inducible deletion of Damaged DNA Binding protein 1 (DDB1), an E3 ubiquitin ligase, in hepatocytes. We characterized the molecular mechanism underlying the compensatory activation and the properties of oval cells (OCs) by methods of mouse genetics, immuno-staining, cell transplantation and gene expression profiling. We show that deletion of DDB1 abolishes self-renewal capacity of mouse hepatocytes in vivo, leading to compensatory activation and proliferation of DDB1-expressing OCs. Partially restoring proliferation of DDB1-deficient hepatocytes by ablation of p21, a substrate of DDB1 E3 ligase, alleviates OC proliferation. Purified OCs express both hepatocyte and cholangiocyte markers, form colonies *in vitro*, and differentiate to hepatocytes after transplantation. Importantly, the DDB1 mutant mice exhibit very minor liver damage, compared to a chemical injury model. Microarray analysis reveals several previously unrecognized markers, including Reelin, enriched in oval cells. Here we report a genetic model in which irreversible inhibition of hepatocyte duplication results in HSC-driven liver regeneration. The DDB1 mutant mice can be broadly applied to studies of HSC differentiation, HSC niche and HSCs as origin of liver cancer.

## Introduction

Stem cells can self-renew, differentiate, and maintain tissue homeostasis. In highly proliferating tissues such as the skin and the gut, stem cells continuously produce transit-amplifying cells that differentiate and replenish short-lived mature cells [1], [2]. By contrast, the liver has very low levels of cell turnover, and it primarily relies on replication of highly differentiated parenchymal hepatocytes to regenerate in response to loss of liver mass [3], [4], [5]. However, when hepatocyte replication is inhibited or compromised, quiescent HSCs can be activated to produce a large number of transit-amplifying progenitor cells (called OCs in rodents), which eventually differentiate to hepatocytes and bile duct cholangiocytes to restore the liver to its original volume [6], [7], [8].

Conventionally, HSC activation and oval cell proliferation are induced in chemical injury rodent models [6], [9], [10]. One of the commonly used methods is the 2-acetylaminofluorene (2-AAF)/partial hepatectomy (PH) model, in which hepatocyte proliferation is blocked by 2-AAF prior to PH-stimulated liver regeneration [11]. This rodent model is well established in rats, but the same procedure does not induce OCs in mice. Alternative protocols such as 3,5-diethoxycarbonyl-1,4-dihydro-collidine (DDC)-supplemented diets and ethionine-supplemented choline-deficient diets have been developed to induce HSC activation in mice [12], [13]. However, these injury models are not ideal for identifying biomarkers of HSCs or studying mechanisms of HSC activation for several reasons. First, these chemicals damage both hepatocytes and non-parenchymal cells including HSCs and OCs. Second, it is impossible to genetically distinguish damaged existing hepatocytes from newly regenerated hepatocytes from activated HSCs. Finally, promiscuous effects of the chemicals would confound experimental result interpretation. As a result, a better model bypassing these difficulties is desirable.

The molecular signals that activate HSCs are just beginning to be addressed in transgenic mice. Overexpression of TWEAK (TNF-like weak inducer of apoptosis), a TNF-family cytokine, can stimulate oval cell proliferation in mouse liver through binding its receptor Fn-14 [14]. Similarly, hepatocyte-specific deletion of the *neurofibromatosis type 2* (*Nf2*) tumor suppressor gene yields a progressive expansion of oval cells [15]. However, whether and how TWEAK or merlin (encoded by *Nf2*) directly stimulates the oval cell proliferation still need to be established.

We recently demonstrated that hepatocyte-specific deletion of DDB1, an adaptor protein for Cullin4 ubiquitin ligase [16], permanently blocks the hepatocytes from self-renewing, resulting in compensatory regeneration of new DDB1-expressing hepatocytes and progressive tumor development [17]. Here we report that one possible source of these new hepatoctyes are the DDB1-expressing oval cells, which are activated in response to the proliferative defect of DDB1-depleted hepatocytes. These DDB1 mutant mice show minor liver damage compared with those chemically injured mice. We have explored this model of HSC activation and identified several candidate markers of oval cells that have not been unraveled using chemical injury models.

## Materials and Methods

### Animals

Generation and characterization of *DDB1^F/F^* and *DDB1^F/F^*;*Mx1-Cre^+/−^* mice have been described previously [17]. *Alb-Cre* strain, *p21* mutant strain and *Rosa26-lacZ* Cre-reporter strain were purchased from The Jackson Laboratory (Bar Harbor, ME) [18]. Immunocompromised 6-week-old *nu/nu* mice were purchased from the Harlan Laboratories (Indianapolis, IN). The diet containing 0.1% DDC was purchased from Purina TestDiet (Richmond, IN). All animals were maintained in pathogen-free facilities and all experiments followed regulations of the investigators' institutional animal care, with approval ID 08-061 from the Sanford-Burnham Medical Research Institute review committee.

### Immunostaining and Imaging

Tissue samples were dissected from mice and fixed in 4% (w/v) paraformaldehyde, embedded in paraffin, and immunostaining were performed as described [19]. The following primary antibodies were diluted in Antibody Diluent with Background Reducing Components (Dako, Denmark) and incubated at 4°C overnight: DDB1 (Bethyl Laboratories, Burlingame, CA), EpCAM (epithelial cell adhesion molecule) (Abcam, Cambridge, MA), A6 (gift from Dr. V. Factor), Cytokeratin19 (CK19) (gift from Dr. R. Oshima), Albumin (Novus Biologicals, Littleton, CO), α-fetoprotein (AFP) (Santa Cruz Biotechnology, Santa Cruz, CA), CD133 (eBioscience, San Diego, CA), ß-galactosidase (Mirus Bio Corporation, Madison, WI), CD45 (eBioscience), F4/80 (eBioscience) and Reelin (Abcam).

For immunocytochemical staining, cells were fixed with PBS containing 4% paraformaldehyde at room temperature for 20 minutes and permeabilized and blocked with blocking buffer containing 0.1% Triton X, 1% BSA and 10% goat serum at room temperature for 30 minutes. Cells were then incubated with primary antibodies at 4°C overnight, followed by secondary antibodies at room temperature for 30 minutes.

### Western Blotting

Hepatocytes isolated after perfusion were lysed in RIPA buffer and lysates were centrifuged at 12,000 rpm for 15 minutes at 4°C to remove cellular debris. Supernatants were diluted in NuPAGE sample buffer (Invitrogen, Carlsbad, CA) and boiled at 70°C for 10 minutes. Protein samples were separated by NuPAGE precast gel (Invitrogen) according to the manufacturer's instruction and transferred to PVDF membranes. The following primary antibodies were incubated overnight at 4°C: p21 (BD Biosciences, Bedford, MA), c-Jun (Santa Cruz Biotechnology), DDB1 (Invitrogen), Lamin B, PCNA and ß-tubulin (Sigma-Aldrich, St. Louis, MO).

### Cell Fractionation

DDB1-deficient mouse embryonic fibroblasts (MEFs) were obtained by infecting primary *DDB1^F/F^* MEFs with adenovirus expressing Cre [19]. Cells were suspended in HB buffer (10 mM Tris, pH8.0, 1.5 mM MgCl_2_, 10 mM KCl, protease inhibitors cocktail) and allowed to swell on ice for 15 min. Triton X-100 (0.2%) was added and the homogenate was centrifuged for 10 min at 1000 g at 4°C. The supernatant (cytoplasmic fraction) was transferred to fresh tube and NaCl concentration was adjusted to 200 mM. Nuclear pellet was washed 5 times with HB buffer containing 0.2% Triton X-100 and resuspended by vortexing at 4°C for 30 minute in buffer C (10 mM Tris, pH8.0, 1.5 mM MgCl_2_, 10 mM KCl, 400 mM NaCl, 0.4% Triton X-100, protease inhibitors cocktail). Homogenate was then centrifuged for 15 min at 20,000 g at 4°C and the nuclear extract was transferred to a fresh tube. Equal volumes of HB buffer were added to bring the NaCl concentration to 200 mM.

### Liver Cell Isolation

Liver cells were isolated using a standard three-step protocol. Liver perfusion was initiated by administering through the portal vein 200 ml of 0.5 M EGTA solution in basic liver perfusion buffer (30 mM KCl, 1.3 M NaCl, 10 mM NaH_2_PO_4_.2H_2_O, 100 mM Glucose and 100 mM HEPES, adjusted to pH7.4 at 37°C). The liver was then washed with 200 ml of basic liver perfusion buffer alone. Subsequently, 0.02% collagenase type 4 (Sigma-Aldrich) and 5 mM CaCl_2_ were added to the basic liver perfusion buffer and perfusion was continued until digestion was complete. The digested liver was suspended in 50 ml of PBS and the dissociated cells were passed through a 100 µm nylon mesh and centrifuged at 50 g for 5 minutes at 4°C. After centrifugation, precipitated cells were used as hepatocyte fraction, and 35 ml of supernatant was transferred to a new tube and washed with PBS. Cells were centrifuged at least twice at 2000 rpm for 5 minutes and the pellets were suspended in ice-cold PBS containing 10% FBS and used as non-parenchymal cells.

### Flow Cytometry Analysis and Sorting

Aliquots of non-parenchymal cells were incubated with red blood cell lysis buffer (Sigma-Aldrich) to eliminate erythrocytes. After washing with PBS, cells were blocked with 10% rat serum (eBioscience) in PBS for 30 minutes on ice. Cells were then incubated with fluorescence-conjugated EpCAM (eBioscience) and F4/80 (eBioscience) antibodies in 3% BSA/PBS for 30 minutes on ice, sorted using FACSVantageSE DiVa (BD Biosciences), and analyzed using FlowJo software.

### Culture of Sorted Cells

EpCAM-positive (EpCAM^+^) cells sorted from non-parenchymal liver cells were suspended in Williams' medium E supplemented with 10% FBS, 2 mM L-glutamine, 100 nM dexamethasone, 1×ITS (insulin, transferrin, selenium X), 10 ng/ml human epidermal growth factor (hEGF), and 10 ng/ml human hepatocyte growth factor (hHGF). Cells were seeded on Matrigel (BD Biosciences) and cultured in 37°C CO_2_ incubator.

### RNA Extraction and Quantitative Real-time Reverse Transcription PCR (RT-PCR)

Total RNA was extracted using RNeasy Mini Kit (QIAGEN, Germantown, MD) according to the manufacturer's instruction. Total RNA and oligo dT primer were used to synthesize cDNA using the Superscript III RT (Invitrogen). Quantitative real-time RT-PCR was performed using the MX3000 Real-Time PCR System (Stratagene, Santa Clara, CA). PCR primers for mouse genes are listed in Table S1. Target cDNAs were normalized to endogenous mRNA levels of the housekeeping reference gene *18s ribosomal RNA* (*18s rRNA*). The reproducibility of this quantification method was examined by comparing results obtained from replicate samples during the same reaction run with those from independent runs on different days. The PCR reactions were performed at least 3 times for each sample. Data are presented as means and standard errors of the mean (SEMs). Statistical significance was assessed with Student *t* test.

### Microarray

Sample amplification, labeling and hybridization on Illumina GeneChip Mouse Genome 48K Arrays were performed for all arrays in this study according to the manufacturer's instructions (Illumina, Inc., San Diego, CA) using an Illumina BeadStation in the Burnham Institute's Microarray Core Facility. Total RNA samples were prepared in RNeasy Mini Kit (QIAGEN) from isolated EpCAM^+^ cells from *DDB1^F/F^;Alb-Cre^+/+^* or DDC-treated wild type liver, and compared with hepatocytes isolated from wild type liver. Data analysis was done in three stages. First, expression intensities were calculated for each gene probed on the array for all hybridizations using the algorithm supplied with the illumina GenomeStudio software. Second, intensity values were quality controlled and normalized: quality control was carried out by using the detection P-value with a cutoff of 0.05. Genes with a P-value only ever above this were removed from the analysis. All the arrays were then normalized using the bioconductor *normalize. quantiles*. This procedure accounted for any variation in hybridization intensity between the individual arrays. These normalized data were imported into GeneSpring and analysed for differentially expressed genes on the basis of fold change (>2×). Microarray data were deposited in the GEO database (accession number GSE31588) and followed MIAME requirements.

### Liver Function Assays

Blood samples collected from mice were analyzed using a mammalian liver enzyme profile rotor on a VetScan VS2 analyzer (Abaxis, Union City, CA).

### Statistical analysis

Differences in real-time PCR data were analyzed by one-way ANOVA followed by Bonferroni post-hoc test. GraphPad Prism software was used for statistical analysis of these data. *P*<0.05 and *P*<0.01 were considered as statistically significant and highly significant respectively.

## Results

### Hepatocyte-specific deletion of DDB1 induces ductal cell proliferation

**Figure 1:**
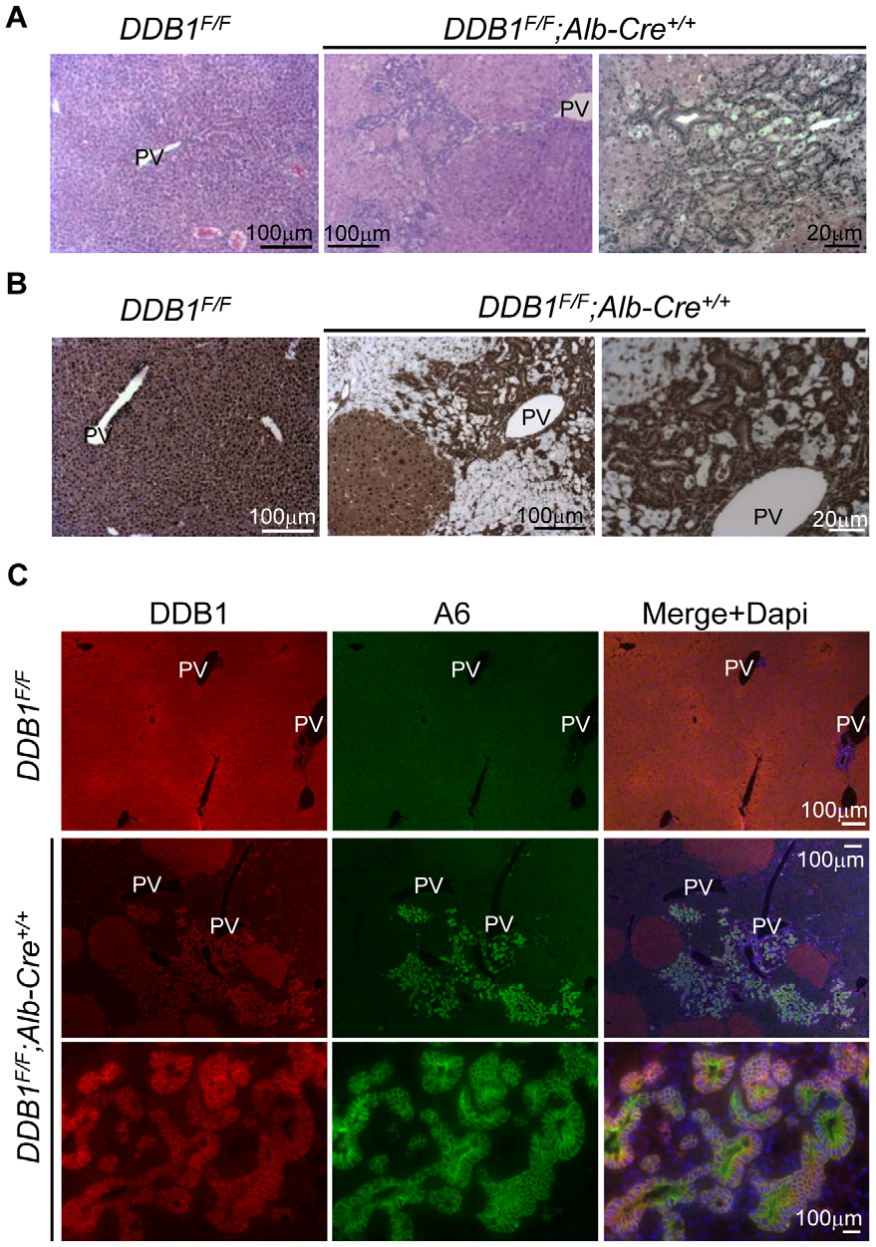
Deletion of DDB1 in hepatocytes results in hepatic oval cells activation. (A) H&E staining of liver sections from 4-week old *DDB1^F/F^* and *DDB1^F/F^;Alb-Cre^+/+^* mice. PV, portal vein. (B) IHC staining for DDB1 on liver sections from 4-week old *DDB1^F/F^* and *DDB1^F/F^;Alb-Cre^+/+^* mice. (C) Co-IF staining for DDB1 and A6 on 4-week old *DDB1^F/F^* and *DDB1^F/F^;Alb-Cre^+/+^* liver sections.

We previously reported that DDB1 is required for hepatocyte replication, and hepatocyte-specific deletion of DDB1 leads to regeneration of new DDB1-expressing hepatocytes [17]. To determine the origin of these regenerated DDB1-expressing hepatocytes, we generated *DDB1^F/F^;Alb-Cre^+/+^* mouse, in which homozygous copies of *Alb-Cre* would allow for maximal Cre expression and thus more effective deletion of DDB1. We found that the liver from *DDB1^F/F^;Alb-Cre^+/+^* mice appeared pale with rough surface between 2 and 3 weeks of age, but small pink regenerative nodules began to emerge from white patches around 4 weeks of age (Figure S1A). H&E staining of the liver sections from 4-week-old mutants revealed massive periportal expansion of small cells that form interconnected ductal structures (Figure 1A). IHC staining for DDB1 demonstrated that DDB1-expressing small cells and hepatocytes are embedded in numerous DDB1-depleted hepatocytes (Figure 1B), after nearly complete lose of DDB1 expression in hepatocytes by three weeks of age (Figure S1B). These DDB1-expressing small cells are generally located adjacent to the portal veins and multiply (Figure S1C), and can be found in the liver from 3-week-old mutant mice prior to the appearance of DDB1^+^ hepatocyte nodules.

### DDB1-expressing small cells in the liver are oval cells

**Figure 2:**
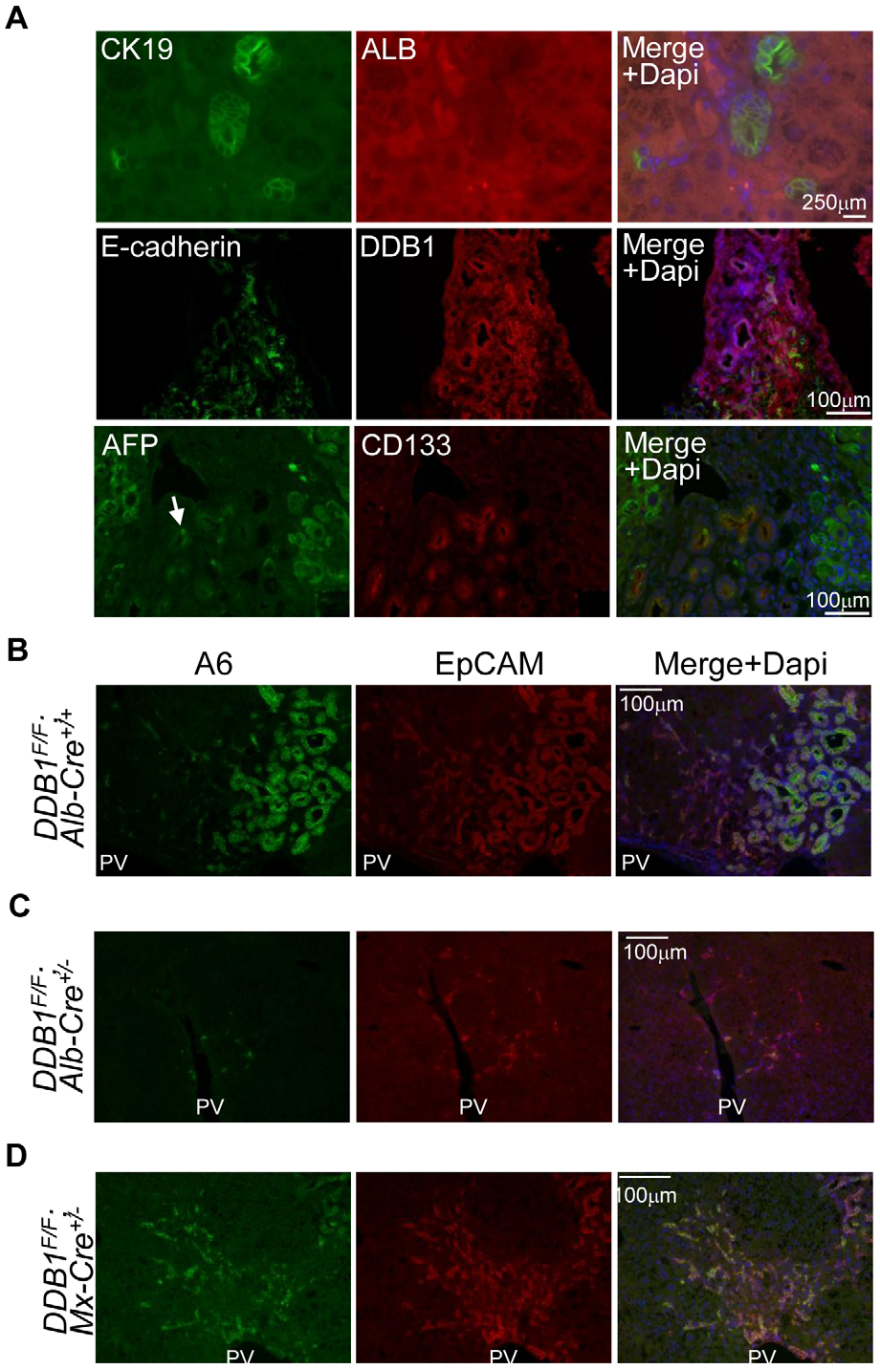
Expression of oval cell markers in DDB1 mutant mouse liver. (A) Co-IF staining for Cytokeratin-19 (CK19) and Albumin (Alb) (upper panels), E-cadherin and DDB1 (middle panels), α-fetoprotein (AFP) and CD133 (lower panels). (B–D) Co-IF staining for EpCAM and A6 on liver sections from 4-week old *DDB1^F/F^;Alb-Cre^+/+^* (B), 6-week old *DDB1^F/F^;Alb-Cre^+/−^* (C), and adult *DDB1^F/F^;Mx1-Cre^+/−^* mice at 6 weeks after receiving poly(I:C) injection (D).

Failure of hepatocyte proliferation or severe liver damage results in activation of HSCs and OCs [9]. OCs are believed to take the form of the ductal reaction and are able to generate new hepatocytes to replace damaged hepatocytes. Morphologically DDB1-expressing ductal cells in *DDB1^F/F^;Alb-Cre^+/+^* liver resemble OCs, with oval nuclei and scant cytoplasm content. To verify that these cells are OCs, we performed IF staining for the OC biomarker A6 [20], [21], and found that these DDB1-expressing ductal cells co-expressed A6 (Figure 1C). Generally these cells appeared adjacent to the portal veins and infiltrated the parenchyma surrounding the DDB1-positive regenerative nodules. In contrast, cells expressing A6 in *DDB1^F/F^* liver were rare and restricted to normal bile duct epithelial cells. These ductal cells from the DDB1 mutant mice expressed a cholangiocyte marker Ck19 but not hepatocyte marker Albumin (Figure 2A). To identify the cell surface molecules expressed on these cells, we performed staining for additional stem or progenitor cell markers [22]. We found that these cells are positive for E-cadherin, CD133 and some cells are positive for AFP (Figure 2A). Taken together, these observations suggest that deletion of DDB1 in hepatocytes induces OC proliferation, judged from these cells' morphology, physical location and biomarker expression.

### Oval cells are activated in other DDB1 mutant mouse strains

EpCAM is expressed in cholangiocytes but not in hepatocytes in normal liver, and it is a well-established marker for proliferating OCs [23]. To determine whether EpCAM is expressed in our mouse model, we performed EpCAM and A6 double IF staining on liver sections from 4 week-old *DDB1^F/F^;Alb-Cre^+/+^* mice. We found that numerous A6-positive ductal cells were also positive for EpCAM (Figure 2B). Less prominent activation of A6*^+^* EpCAM*^+^* OCs was observed in the liver of *DDB1^F/F^;Alb-Cre^+/−^* mice at 6 weeks of age, when DDB1-expressing hepatocytes are actively replacing DDB1-deficient hepatocytes (Figure 2C) [17]. An inducible model, *DDB1^F/F^;Mx1-Cre^+/−^* adult mice [17] also exhibited numerous ductal cells expressing both A6 and EpCAM when new hepatocytes were regenerated 6 weeks after receiving injection of poly(I:C) (Figure 2D). Taken together, these findings indicate that both constitutive and inducible DDB1 deletion in hepatocytes results in OC proliferation.

### Deletion of p21 partially restores proliferation of DDB1-deficient hepatocytes and alleviates OC activation

**Figure 3:**
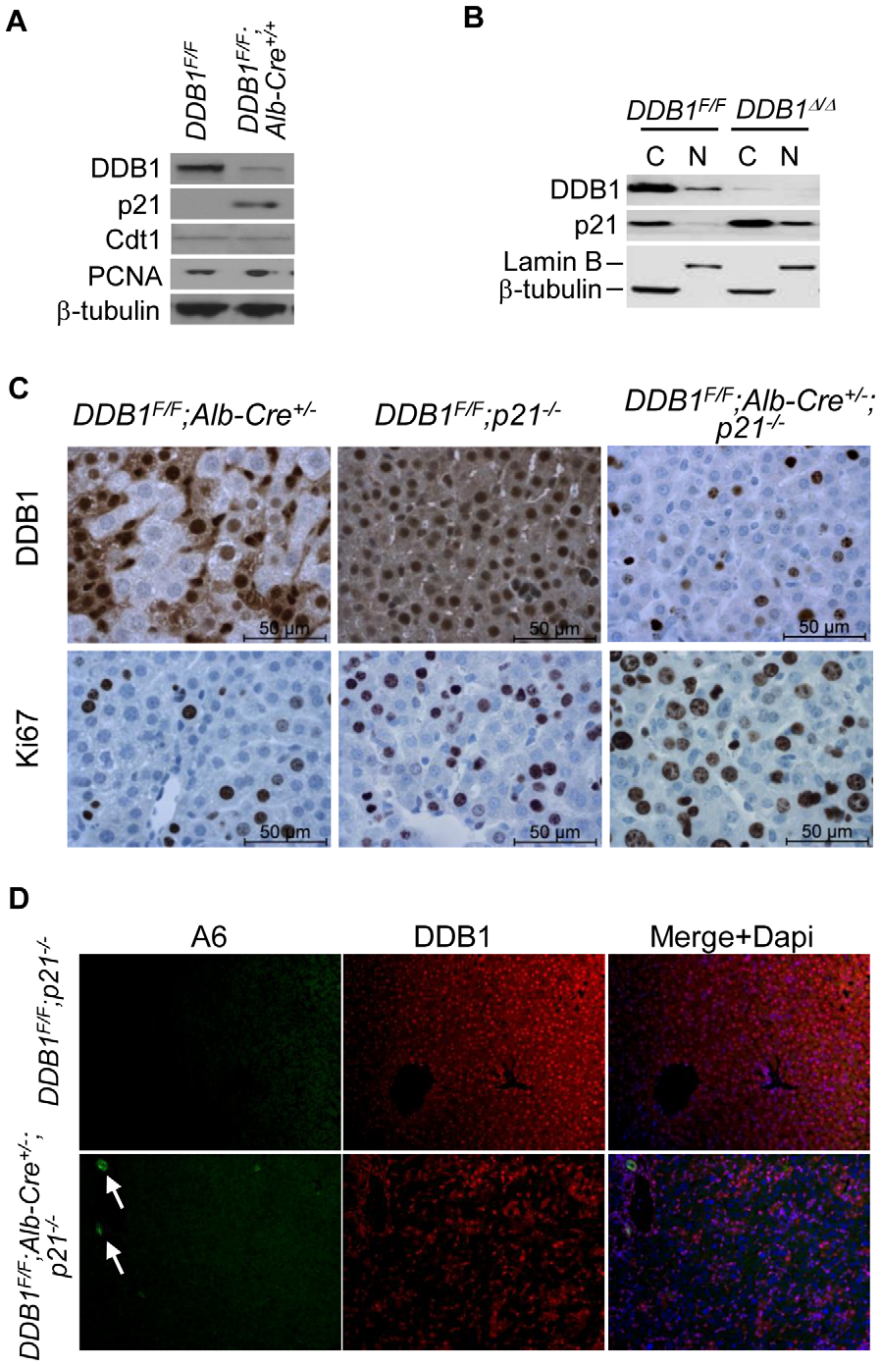
Deletion of p21 partially restores proliferation of DDB1-deficient hepatocytes and alleviates OC activation. (A) Western blot for some substrates of DDB1-Cul4A ubiquitin ligase using lysates of hepatocytes isolated from *DDB1^F/F^* and *DDB1^F/F^;Alb-Cre^+/+^* mice. (B) Western blot for DDB1 and p21 using lysates of cytoplasmic fraction (C) and nuclear fraction (N) prepared from MEFs. (C) IHC staining for DDB1 and Ki67 on liver sections from *DDB1^F/F^;Alb-Cre^+/−^, DDB1^F/F^;p21^−/−^* and *DDB1^F/F^;Alb-Cre^+/−^;p21^−/−^* mice. (D) Co-IF staining for A6 and DDB1 on *DDB1^F/F^;p21^−/−^* and *DDB1^F/F^;Alb-Cre^+/−^;p21^−/−^* liver sections. Arrows in *DDB1^F/F^;Alb-Cre^+/−^;p21^−/−^* mice indicate A6 positive oval cells.

We previously reported that DDB1-deficient hepatocytes accumulate cell cycle inhibitor p21, a substrate of DDB1 ubiquitin ligase [24], [25], and fail to proliferate during liver growth or after partial hepatectomy [17]. Similarly, hepatocytes from *DDB1^F/F^;Alb-Cre^+/+^* mice express high levels of p21, but not Cdt1 (Figure 3A), another substrate of the E3 ligase [16]. p21 is dramatically enriched in the nuclear fraction of DDB1-depleted MEFs [19] (Figure 3B), consistent with a role of p21 in blocking proliferation of these cells. Indeed, deletion of p21 abrogates the cell cycle arrest of cells with inactivated Cul4A-DDB1 ubiquitin ligase [25], [26]. We therefore generated compound mutant mice by crossing *DDB1^F/F^;Alb-Cre^+/−^* mice and *p21^−/−^* mice [27]. Deletion of p21 in *DDB1^F/F^;Alb-Cre^+/−^* mice allowed DDB1-decificent hepatocytes to proliferate (Figure 3C), resulting in significantly less compensatory proliferation of DDB1-expressing OCs (Figure 3D). However, p21 deletion did not fully reverse the proliferative arrest of DDB1-deficient hepatocytes (data not shown). These hepatocytes may still have other defects such as epigenetic deregulation due to the loss of the DDB1Cul4 ubiquitin ligase activity [28], [29], [30]. We conclude that failed proliferation of DDB1-deficient hepatocytes is responsible for activation of DDB1-expressing OCs.

### EpCAM-positive cells in DDB1 mutant form colonies in vitro and repopulate the liver in vivo

**Figure 4:**
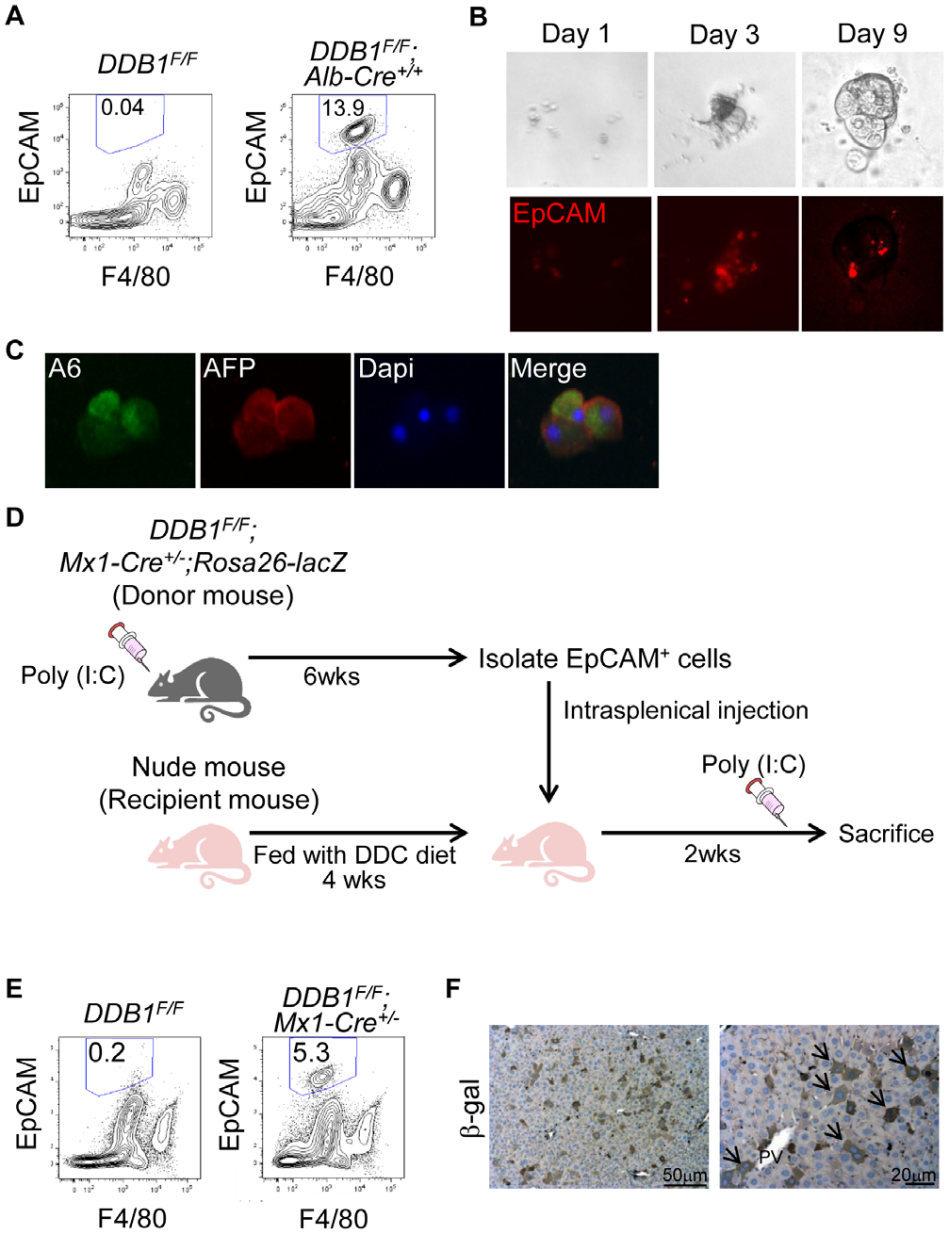
EpCAM-positive cells from DDB1 mutant liver proliferate *in vitro* and repopulate the liver *in vivo*. (A) Analysis by flow cytometry of EpCAM^+^ F4/80^−^ cells in non-parenchymal fractions prepared from 4-week old *DDB1^F/F^* and *DDB1^F/F^;Alb-Cre^+/+^* mice. The ratio of EpCAM^+^ F4/80^−^ cells from a representative experiment is shown in each panel. (B) *In vitro* culture of EpCAM^+^ cells sorted by FACS from DDB1 mutant liver and seeded on Matrigel. Cells form colonies after 1, 3, and 9 days of culture (upper panels) and exhibit EpCAM positivity (lower panels). (C) Co-IF staining for A6 and AFP on colonies from cultured EpCAM^+^ cells. (D) Experimental scheme for *in vivo* differentiation of EpCAM^+^ OCs. Adult *DDB1^F/F^;Mx1-Cre^+/−^;Rosa26-lacZ* mice were generated and injected with poly(I:C). EpCAM^+^ cells were isolated by FACS 6 weeks later, and injected intrasplenically to nude mice that had been fed with DDC diet. The recipient nude mice received poly(I:C) injection 2 weeks later and liver collected for analysis on the following day. (E) Isolation of EpCAM^+^ F4/80^−^ cells from donor or control mouse liver. The ratio of EpCAM^+^ F4/80^−^ cells is shown in each panel. (F) IHC staining for ß-gal on liver sections from recipient nude mice. Arrows indicate positively stained hepatocytes.

To characterize these OCs, we perfused the liver of *DDB1^F/F^;Alb-Cre^+/+^* mice, separated non-parenchymal cells from hepatocytes, and isolated EpCAM^+^ and F4/80^−^ cells by FACS. We typically harvested around 13% of EpCAM^+^ cells from the non-parenchymal cell fraction prepared from the mutant liver, but harvested barely any such cells from age-matched wild type liver (Figure 4A). When cultured on Matrigel-coated plates, these cells formed colonies and retained EpCAM expression (Figure 4B). Even after one month in culture, cells in the colonies still express A6 and AFP (Figure 4C), resembling those OCs in vivo.

Regeneration of new hepatocytes to repair injured liver is a pivotal function for hepatic stem/progenitor cells. It was previously reported that OCs isolated from mouse liver could reconstitute hepatic tissues after transplantation into mice with injured liver [13], [31]. To determine whether OCs isolated from DDB1 mutant liver can give rise to new hepatocytes *in vivo*, we crossed *DDB1^F/F^;Mx1-Cre^+/−^* mice with a *Rosa26-lacZ* Cre-reporter mouse strain [18]. We isolated EpCAM^+^ cells by FACS from *DDB1^F/F^;Mx1-Cre^+/−^*;*Rosa26-lacZ* mice that had been injected with poly(I:C), and then injected these cells intrasplenically into nude mice (Figure 4D, E). Prior to cell transplantation, the recipient nude mice had been preconditioned with DDC diet for 4 weeks to block hepatocyte proliferation. The nude mice were sacrificed 2 weeks after injection of EpCAM^+^ cells and, one day before sacrificing, received one i.p. injection of poly(I:C) to activate LacZ expression specifically from donor cells. IHC staining on these liver sections showed many ß-gal-positive hepatocytes (Figure 4F), suggesting that the transplanted EpCAM^+^ OCs differentiated into hepatocytes in the recipient liver.

### DDB1 mutant mice exhibit minor liver damage compared with DDC-treated mice

**Figure 5:**
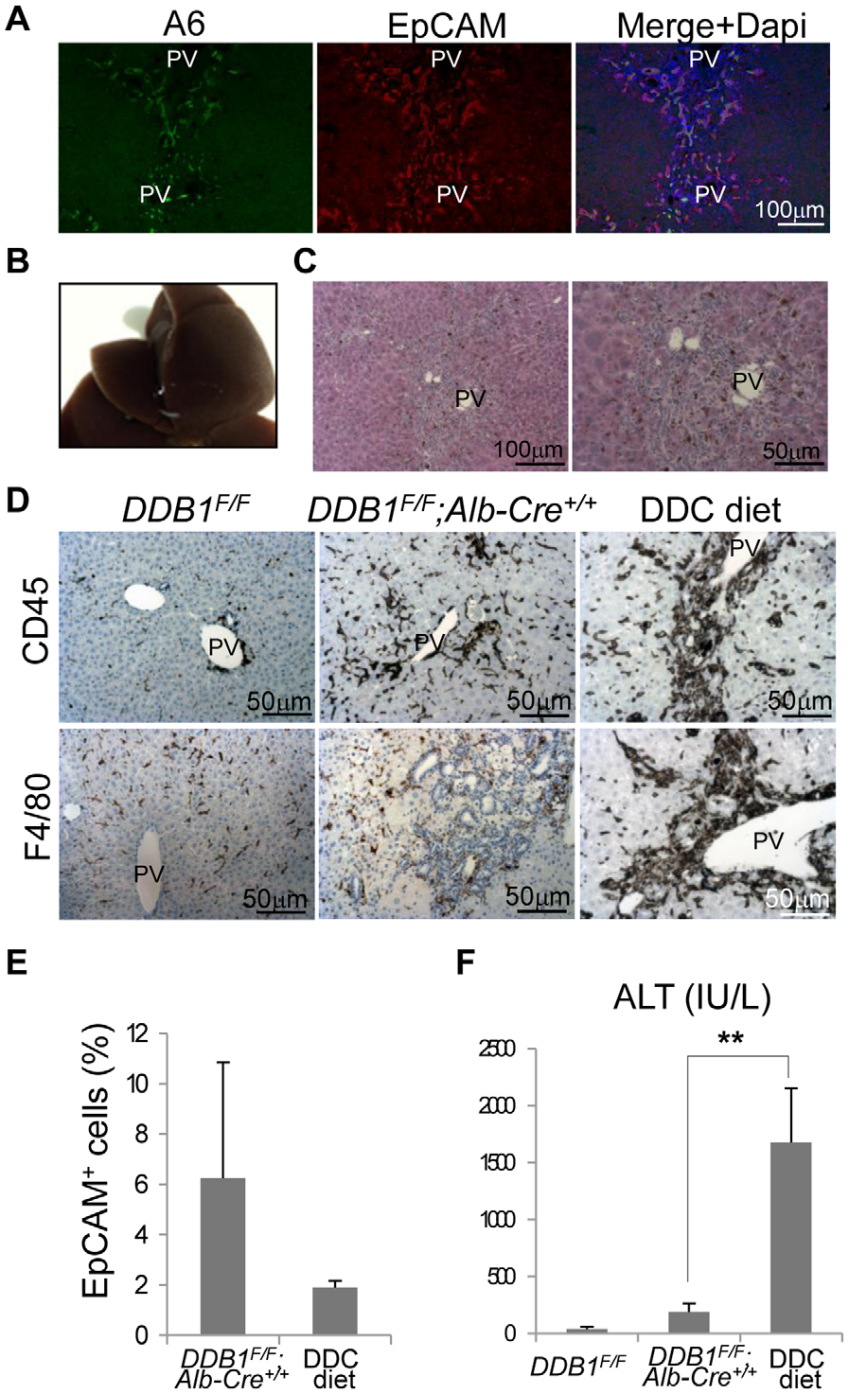
DDB1 mutant mice exhibit minor liver damage compared with DDC-treated mice. (**A**): Co-IF staining for A6 and EpCAM on liver sections from DDC-treated mice. (**B**): Gross appearance of liver from mice fed with DDC diet for 4 weeks. (**C**): H&E staining of the liver in (**B**). (**D**): IHC staining for CD45 (upper panels) and F4/80 (lower panels) on liver sections from *DDB1^F/F^*, *DDB1^F/F^;Alb-Cre^+/+^* and DDC-treated mice. (**E**): Percentage of EpCAM^+^ cells from *DDB1^F/F^;Alb-Cre^+/+^* and DDC-diet liver, determined by FACS analysis. Data are representative of 4 independent experiments with 3 mice per group in each experiment. Values are expressed as the means±SEM; n = 3. (**F**): Serum alanine aminotransferase (ALT) levels. Data are representative of 4 independent experiments with 3 mice per group in each experiment. Values are expressed as means±SEM; n = 3 **P<0.01.

To determine the severity of liver damage in the DDB1 mutant mice, we compared 4-week old *DDB1^F/F^;Alb-Cre^+/+^* mice with a conventional chemical injury model for OC proliferation, mice treated with diet containing 3,5-diethoxycarbonyl-1,4-dihydro-collidine (DDC). Consistent with previous reports [13], [32], [33], ductal cells from DDC-treated wild type mice expressed both A6 and EpCAM (Figure 5A). After treating mice with the diet for 4 weeks, their livers turned dark brown (Figure 5B), due to hepatic porphyria resulting from the inhibition of the heme biosynthetic pathway [34]. H&E staining showed some ductal cells around the portal veins with brown metabolite deposits (Figure 5C). To characterize the extent of liver inflammation, we performed CD45 and F4/80 staining for leukocytes and macrophages, respectively. In liver sections from DDC-treated mice, we found greatly enriched CD45*^+^* and F4/80*^+^* cells surrounding OCs (Figure 5D). In contrast, *DDB1^F/F^;Alb-Cre^+/+^* mouse liver did not show significant increase of these immune cells compared with *DDB1^F/F^* liver (Figure 5D), even though there are more OCs in the DDB1 mutant liver than DDC mice (Figure 5E). We further measured the severity of liver damage by serum alanine aminotransferase (ALT) assays. The ALT level in the DDB1 mutant mice was 191±73 (IU/L); however, it was nearly 10 times higher, reaching 1679±475 (IU/L) (normal range: 28–132 U/L) in the DDC-treated mice (Figure 5F), consistent with the overall toxicity of DDC to the liver. With seemingly insignificant damage to hepatocytes, our genetic model provides a better system for studying HSC and OC biomarkers and their extracellular activation signals.

### Characterization of OCs from DDB1 mutant mice

Since OCs from our DDB1 mutant mice have not been exposed to promiscuous chemical damage, we decided to identify new OC biomarkers that might have been masked in chemical injury models. EpCAM*^+^* cells were isolated from *DDB1^F/F^;Alb-Cre^+/+^* mice and from DDC-treated mice, and their gene expression profiles were analyzed with Illumina genechips (Table S2). Wild type hepatocytes were purified and used as a control. EpCAM^+^ cells from both genetic and chemical models express many known OC markers such as Cldn4, Itgb4, Dmbt1, Ck19, Ncam1, Cd24a, Connexin43 [35], [36]. These cells also express hematopoietic stem cell markers such as Sca-1 and Cd44, but not Cd34 or C-kit. Fn14 receptor, encoded by *Tnfrsf12a*, is upregulated in OCs, agreeing with the reported role of TWEAK/Fn14 in OC proliferation [14]. Recently, Sox-9 was reported as a marker for progenitor cell population and contributes to the self renewal and repair of the adult liver [37]. We found Sox-9 is also highly upregulated in OCs.

**Figure 6:**
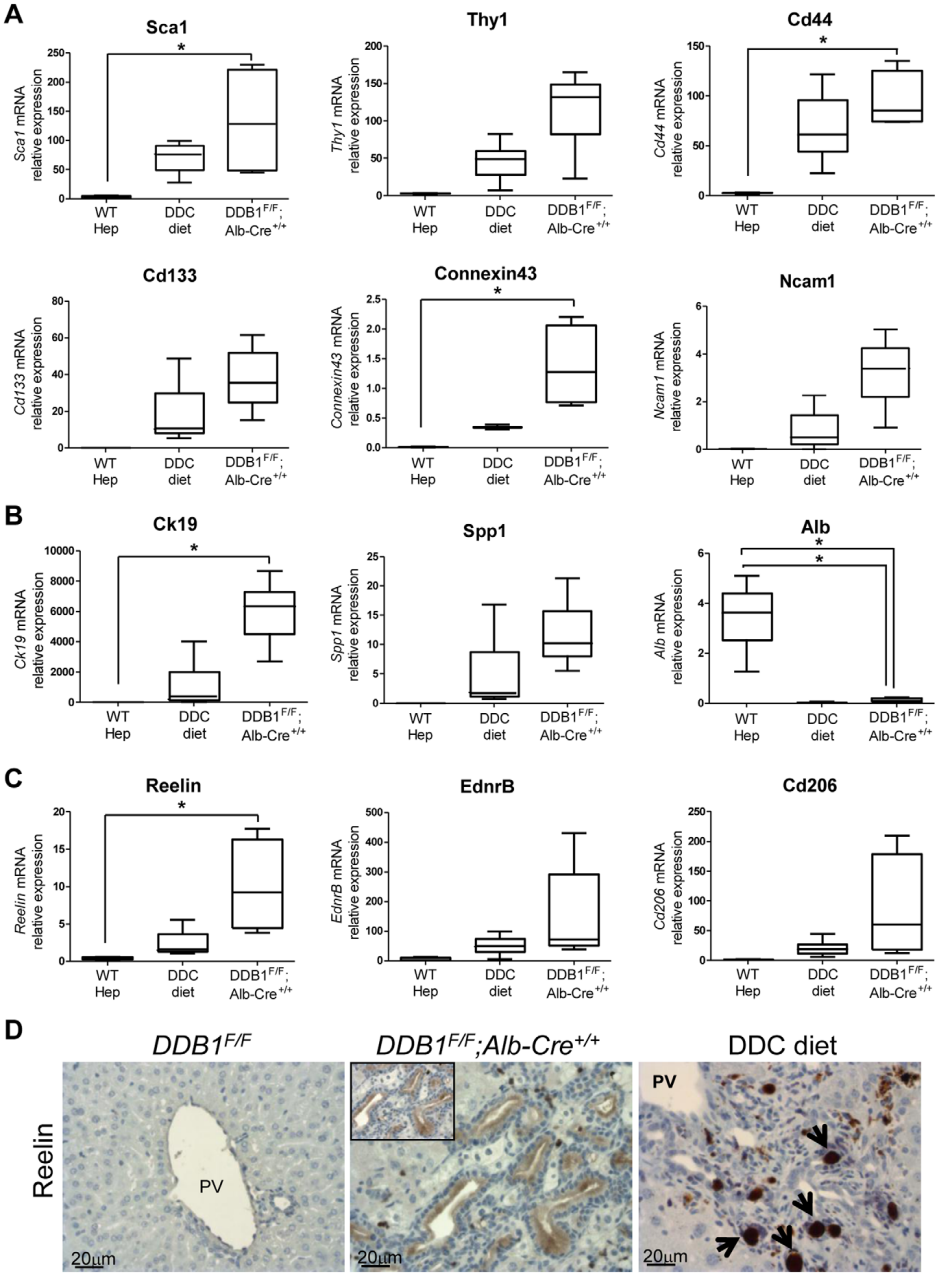
Characterization of OCs from DDB1 mutant mice and DDC-treated mice. (**A–C**): Quantitative real-time PCR analysis for selected genes expressed in hepatocytes isolated from wild type mice, and EpCAM^+^ cells isolated from DDC-treated and *DDB1^F/F^; Alb-Cre^+/+^* mice. All data are normalized to *18s* rRNA level. Data are representative of 4 independent experiments with 3–4 mice per group. *P<0.05 (**A**): Expression levels of hematopoietic markers *Sca1*, *Thy1* and *Cd44*, and OC markers *CD133*, *Connexin43* and *Ncam1*. (**B**): Expression levels of cholangiocyte marker *Ck19* and *Spp1*, and hepatocyte marker *Alb*. (**C**): Expression levels of candidate OC markers, *Reelin*, *EdnrB* and *Cd206.* (**D**): IHC staining for reelin on liver sections from *DDB1^F/F^*, *DDB1^F/F^;Alb-Cre^+/+^* and DDC-treated mice. Arrows in DDC-treated liver indicate non-specific brown deposits.

We performed real-time RT-PCR to validate the expression profiling. Expression levels of candidate genes including *Sca1, Thy1, Cd44, CD133, Connexin43* and *Ncam1* are all confirmed to be increased (Figure 6A) [35], [38], [39]. As expected, OCs from both models expressed cholangiocyte markers (*Ck19, Spp1*), but not hepatocyte marker (*Alb*) (Figure 6B). Therefore, OCs from the DDB1 mutant mouse model are generally similar to the cells isolated from a conventional injury model. Though many genes are upregulated in both models, several previously unrecognized genes have been found to be upregulated only in OCs from DDB1 mutant mice. To search for new stem/progenitor markers masked in chemical models, we analyzed genes that are specifically enriched in OCs isolated from the DDB1 mutant liver in comparison with OCs from DDC-treated liver. Three candidate genes, *Reelin*, endothelin receptor type B (*EdnrB*), *Cd206* (also known as *MRC1*, mannose receptor, C type 1) were identified and confirmed by quantitative real-time PCR (Figure 6C). Reelin, an extracellular matrix protein, controls neuronal migration during embryonic brain development [40]. Reelin was reported to be expressed in activated hepatic stellate cells [41]. We found Reelin was abundant in OCs in DDB1 mutant liver (Figure 6D). Further experiments are required to determine biomarker or functional properties of Reelin and the other 2 candidate genes in HSC activation and OC proliferation.

## Discussion

It has been claimed for decades that HSCs can be activated and contribute to liver regeneration when hepatocyte proliferation is compromised. Supports from this claim mostly come from studies of rodents treated with chemicals that intoxicate the liver. Here we provide direct evidence that targeted deletion of DDB1 in hepatocytes blocks replication of these cells and as a consequence quiescent HSCs and OCs proliferate in a compensatory regeneration process. Unlike chemicals, genetic ablation of DDB1 is restricted to hepatocytes and leaves intact non-parenchymal cells including HSCs and OCs. Therefore this model is ideal for dissecting the extracellular signals that mediate the cross-talks between damaged hepatocytes and activated HSCs. For example, our array data indicate that Fn14, a TWEAK receptor, is up-regulated in EpCAM*^+^* OCs. Our next step will be to determine the cellular origin of TWEAK, either being DDB1-deficient hepatocytes or being newly regenerated DDB1-expressing hepatocytes.

Since we have not performed genetic tracing on these models, we cannot conclude that all DDB1-positive hepatocytes come from HSCs. Though deletion of DDB1 is quite complete, we cannot rule out that a few hepatocytes escape Cre-mediated deletion and thus contribute to the pool of new hepatocytes. However, it is clear that initial hepatocyte proliferation is essential for HSCs activation. Interestingly, completely blocking regeneration of DDB1-expressing hepatocytes, which can be achieved by frequent injections of poly(I:C) to *DDB1^F/F^;Mx1-Cre^+/−^* mice results in almost no activation of OCs (Endo and Cang, unpublished data). This observation suggests that newly regenerated hepatocytes might provide paracrine factors critical for either activation of HSCs or proliferation of OCs.

Another advantage of the DDB1 model over the injury model is that these genetic mutant mice exhibit much milder liver damage during the course of HSC-driven liver regeneration, although both models produce OCs expressing similar markers (Fig. 5) that are also reported in OCs from other models [35], [42], [43]. We therefore performed microarray analysis of EpCAM^+^ oval cells isolated from the DDB1 mutant mice, and hoped to unravel novel biomarkers that are suppressed in the severely damaged liver of conventional injury rodents. Indeed, we identified three candidate genes, *Reelin*, *EdnrB*, and *Cd206*, which are up-regulated only in OCs from the DDB1 mutant mice. These genes were not expressed in OCs from the injury models and have not been reported as OC markers. Whether these genes have functional roles in HSC activation and OC proliferation or simply are biomarkers still awaits investigation.

We present two mouse genetic models that bypass the hurdles to work with the prevailing injury models. In *DDB1^F/F^;Mx1-Cre^+/−^* mice, HSCs can be repeatedly induced by poly(I:C) injection to generate transit-amplifying oval cells and new hepatocytes, both expressing DDB1 and therefore clearly distinct from DDB1-depleted old hepatocytes. In *DDB1^F/F^;Alb-Cre^+/−^* or *DDB1^F/F^;Alb-Cre^+/+^* mice, DDB1-depeleted hepatocytes are continuously replaced by new DDB1-expressing hepatocyte generated by HSCs. The high rate of hepatocyte turnover driven by HSCs in the mutant liver, in contrast to a very slow turnover rate in normal liver [37], is reminiscent of highly proliferative tissue such as skin and intestine in which stem cells continually generate progeny with diminished replicative capacity. These two models will allow us to trace the origin and differentiation of HSCs as well as perform genetic assessment of signalling pathways important for regulating this mysterious population of cells in the liver.

## Supporting Information

Figure S1 **Deletion of DDB1 in hepatocytes results in ductal cell proliferation.** (A) Gross appearance of livers dissected from 4-week old *DDB1^F/F^* and *DDB1^F/F^;Alb-Cre^+/+^* mice. (B) IHC staining for DDB1 on liver sections from 3-week old *DDB1^F/F^;Alb-Cre^+/+^* mice. Arrows indicate DDB1-positive small cells. (C) DDB1 positive ductal cells (left panel) express Ki-67 (right panel).
(TIF)

Table S1 **Oligonucleotides used in real time RT-PCR.**
(DOCX)

Table S2 **Gene Expression of hepatic oval cells.**
(DOCX)
