# Supplementary figures and images for: Genetic Abolishment of Hepatocyte Proliferation Activates Hepatic Stem Cells
Source: PLoS One. 2012 Feb 23;7(2):e31846. doi: 10.1371/journal.pone.0031846 (PMC3285627; doi:10.1371/journal.pone.0031846)

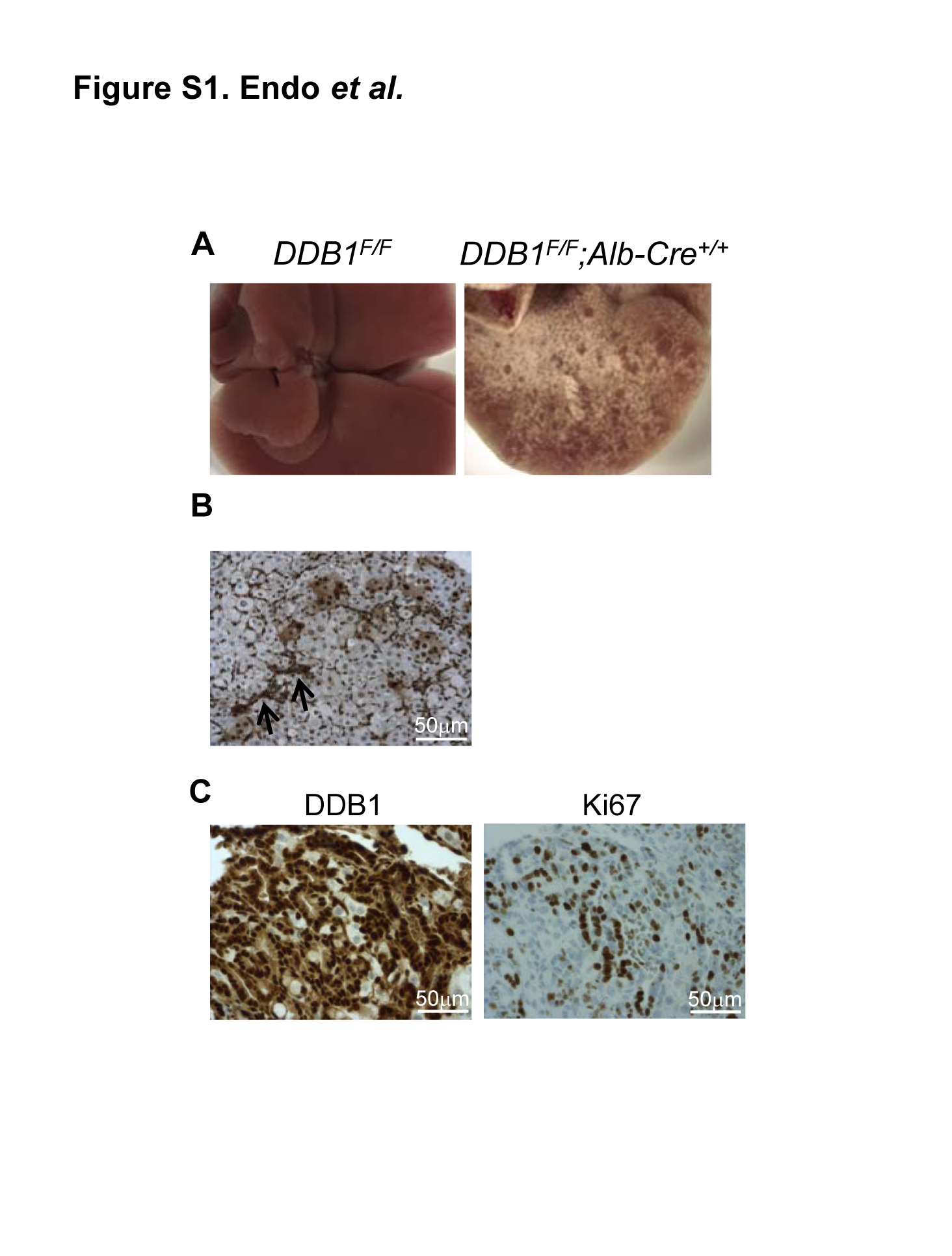

Supplement: Figure S1 — Deletion of DDB1 in hepatocytes results in ductal cell proliferation. (A) Gross appearance of livers dissected from 4-week old DDB1F/F and DDB1F/F;Alb-Cre+/+ mice. (B) IHC staining for DDB1 on liver sections from 3-week old DDB1F/F;Alb-Cre+/+ mice. Arrows indicate DDB1-positive small cells. (C) DDB1 positive ductal cells (left panel) express Ki-67 (right panel). (TIF) [file pone.0031846.s001.tif]
