# Supplementary material for: Genetic Abolishment of Hepatocyte Proliferation Activates Hepatic Stem Cells
Source: PLoS One. 2012 Feb 23;7(2):e31846. doi: 10.1371/journal.pone.0031846 (PMC3285627; doi:10.1371/journal.pone.0031846)
Supplement: Table S1 — Oligonucleotides used in real time RT-PCR. (DOCX) [file pone.0031846.s002.docx]

**Table S1.　Oligonucleotides used in real time RT-PCR**

| Gene | Forward (5’ to 3’) | Reverse (3’ to 5’) |
| --- | --- | --- |
| *Alb* | TCCTGATTGCCTTTTCCCAGTATCT | GCCAGTTCACCATAGTTT TCACGGA |
| *Ck19* | ACTTGCGCGACAAGATTC | AACTTGGTTCTGAAGTCATCTGC |
| *Spp1* | GGCATTGCCTCCTCCCTC | GCAGGCTGTAAAGCTTCTCC |
| *Sca1* | CAATTACCTGCCCCTACCT | CCTTAGTACCCAGGATCTCCATAC |
| *Cd44* | CACATATTGCTTCAATGCCTCAG | CCATCACGGTTGACAATAGTTATG |
| *Thy1* | GGTCCTTACCCTAGCCAACT | GCAGCATCCAGGATGTGTTC |
| *Cd133* | TCATCGCTGTGGTCGTCATTG | GTCCGCTGGTGTAGTGTTGTAG |
| *Ncam1* | CCATCACGGTTGACAATAGTTATG | CAGCGGTAAGTACCCTCATC |
| *Connexin43* | GATTGAAGAACACGGCAAGG | AGAGCGAGAGACACCAAGGA |
| *Afp* | TCAAGAACTCACCCCAACCT | GGCTCTCCTCGATGTGTTTC |
| *Reelin* | CAACACCAGCAAAGTATGATG | TTTAAGCATGGGCTTTTAGC |
| *Ednrb* | ACAAAGGAAAGCCCCTAA | ATAAAAGACTGCAGTGATGG |
| *Cd206* | GCCAGAGACATAACAGCA | CAGGTTTCCTTTCAGTCCT |
| *Lgr5* | GACAATGCTCTCACAGAC | GGAGTGGATTCTATTATTATGG |
| *18s* | GTA ACCCGTTGAACCCCA TT | CCATCCAATCGGTAGTAGCG |
