# Supplementary material for: Genetic Abolishment of Hepatocyte Proliferation Activates Hepatic Stem Cells
Source: PLoS One. 2012 Feb 23;7(2):e31846. doi: 10.1371/journal.pone.0031846 (PMC3285627; doi:10.1371/journal.pone.0031846)
Supplement: Table S2 — Gene Expression of hepatic oval cells. (DOCX) [file pone.0031846.s003.docx]

**Table S2. Gene Expression of hepatic oval cells**

| **Symbol** | **Gene Name** | **Fold Change** | | | | | Illumina ID † | |
| --- | --- | --- | --- | --- | --- | --- | --- | --- |
|  |  | DDB1 ^F/F^;Alb-Cre^+/+^* | | | DDB1 ^F/F^;Alb-Cre^+/+^ /DDC diet † | |  |  |
| **Dmbt1** | Deleted in malignant brain tumor | 210.0648 | 3.673422 | | | ILMN_2629239 | | |
| **Cx43** | Connexin43 | 65.4057 | 0.39409 | | | ILMN_1244291 | | |
| **Cldn4** | Claudin4 | 40.25137 | 0.126288 | | | ILMN_1223949 | | |
| **Sox9** | SRY-box containing gene 9 | 37.39314 | 1.399958 | | | ILMN_2778151 | | |
| **Itgb4** | Integrin beta-4 | 31.7937 | 0.328472 | | | ILMN_3144575 | | |
| **Afp** | Alpha fetoprotein | 17.05499 | 7.516231 | | | ILMN_1245472 | | |
| **Cd44** | Cd44 | 16.8391 | 0.340863 | | | ILMN_1223697 | | |
| **Tnfsf12a** | Tumor necrosis factor receptor superfamily, member 12a (Fn14) | 14.28763 | 0.422023 | | | ILMN_2424299 | | |
| **Ednrb** | Endothelin receptor type B | 13.13272 | 7.975042 | | | ILMN_2589640 | | |
| **Ncam1** | Neural cell adhesion molecule 1 | 10.32549 | 0.268095 | | | ILMN_2804166 | | |
| **Muc1** | Mucin 1, transmembrane | 9.503389 | 0.246299 | | | ILMN_2928489 | | |
| **Cldn7** | Claudin7 | 6.703314 | 0.802454 | | | ILMN_2595477 | | |
| **Ck18** | Cytokeratin-18 | 5.798895 | 0.273644 | | | ILMN_2711267 | | |
| **E-cad** | E-cadherin | 5.45863 | 0.814993 | | | ILMN_2628629 | | |
| **Mrc1** | Mannose receptor, C type 1 | 5.12589 | 4.265823 | | | ILMN_1239430 | | |
| **Cd24a** | Cd24a | 3.993515 | 0.245116 | | | ILMN_1237868 | | |
| **Reln** | Reelin | 2.190358 | 26.19373 | | | ILMN_2704257 | | |
| **Dlk1** | Delta-like 1 homolog | 1.375305 | 1.115234 | | | ILMN_2771738 | | |
| **Hepatocyte specific genes** | | | | | | | | |
| **Alb** | Albumin | -7.059133 | 0.96701 | | | ILMN_2651160 | | |
| **Hnf4a** | Hepatocyte nuclear factor 4, alpha | -3.372866 | 0.640368 | | | ILMN_1237767 | | |
| **Cholangiocyte markers** | | | | | | | | |
| **Spp1** | Secreted phosphoprotein 1 | 100.4196 | 0.610311 | | | ILMN_2690603 | | |
| **Ck19** | Cytokeratin-19 | 36.50504 | 0.189504 | | | ILMN_2614462 | | |
| **Hematopoietic markers** | | | | | | | | |
| **Sca1** | Lymphocyte antigen 6 complex, predicted | 13.00222 | 1.542928 | | | ILMN_1255416 | | |
| **Cd44** | Cd44 | 16.8391 | 0.25481 | | | ILMN_1223697 | | |
| **Thy1** | Thymus cell antigen 1, theta | 1.988748 | | 1.054972 | | | | ILMN_2964185 |

* Fold changes in mRNA expression from hepatic oval cells from DDB1^F/F^;Alb-Cre^+/-^

†Fold changes fraction compared between DDB1^F/F^;Alb-Cre^+/-^ and DDC diet mice

††Illumina ID is the gene number in the Illumina gene database.
